# The Yin and Yang of centromeric cohesion of sister chromatids: mitotic kinases meet protein phosphatase 2A

**DOI:** 10.1186/1747-1028-1-9

**Published:** 2006-05-26

**Authors:** Wei Dai, Xiaoxing Wang

**Affiliations:** 1 Division of Molecular Carcinogenesis, Department of Medicine, New York Medical College, Valhalla, NY 10595, USA

## Abstract

Accurate chromosome segregation during meiosis and mitosis is essential for the maintenance of genomic stability. Defects in the regulation of chromosome segregation during division predispose cells to undergo mitotic catastrophe or neoplastic transformation. Cohesin, a molecular glue holding sister chromatids together, is removed from chromosomes in a stepwise fashion during mitosis and meiosis. Cohesin at centromeres but not on chromosome arm remains intact until anaphase onset during early mitosis and the initiation of anaphase II during meiosis. Several recent studies indicate that the activity of protein phosphatase 2A is essential for maintaining the integrity of centromeric cohesin. Shugoshin, a guardian for sister chromatid segregation, may cooperate with and/or mediate PP2A function by suppressing the phosphorylation status of centromeric proteins including cohesin.

## Cohesin and sister chromatid cohesion

Sister chromatid cohesion is established during DNA replication and maintained throughout G_2 _and mitotic prophase and metaphase. At the molecular level, cohesin mediates sister chromatid cohesion through formation of a proposed "ring" structure that entraps chromosomes [1]. The cohesin complex is composed of Smc1, Smc3, Scc1 (substituted by Rec8 in meiosis), and Scc3 (SA1, SA2, or SA3 as an orthologue in vertebrates). During mitosis, cohesin in animal cells is removed from chromosomes in a stepwise fashion through the action of Plk1 and separase, respectively [2,3]. Upon mitotic entry, cohesin dissociates itself along the entire length of chromosomal arms, but not at centromeres, which requires the activity of Plk1 [2]. At anaphase entry, separase activity removes centromeric cohesin [3] that is otherwise inhibited by the spindle checkpoint during prophase and metaphase. Similar temporally-regulated release of cohesin also exists during meiosis. At the first meiotic division (meiosis I), separase cleaves Rec8 along chromosome arms but not at centromeres, resulting in segregation of homologous non-sister chromosomes. During meiosis II, centromeric cohesin is cleaved through the second wave of separase activity, leading to disjunction of sister centromeres and formation of haploid gametes [4,5].

During the past several years, the mechanism preventing centromeric cohesin from undergoing cleavage/dissociation during early mitosis as well as meiosis I and early meiosis II has been a subject of intensive investigation [6,7]. Through the pioneering research efforts of Orr-Weaver and Watanabe groups, a family of proteins termed shugoshins/MEI-S332, has been identified in various eukaryotic model systems [8-10]. This work led to the realization that they play a critical role in mediating protection of centromeric cohesion of sister chromatids during mitosis and meiosis (see a review [11]). Whereas vertebrate shugoshin 1 (Sgo1) functions to protect centromeric cohesin during mitosis [12,13], Sgo1 in yeast guards against premature removal of cohesin at centromeres during meiosis [8,9,14]. However, the exact molecular mechanism by which centromeric cohesin is shielded from dissociation remains unclear even though Sgo1 plays a critical role in protecting sister chromatid cohesion.

## The phosphatase connection

A recent study by the Nasmyth group showed that expression of SA2 mutant resistant to Plk1 phosphorylation results in suppression of premature separation of sister chromatids in Sgo1-depleted cells [12]. This suggests that prevention of cohesin phosphorylation by mitotic kinases such as Plk1 may be a major molecular event through which centromeric cohesion is retained until anaphase entry. There are at least two scenarios that can explain Sgo1-mediated protection of cohesin. (i) Factors such as Sgo1 are recruited to centromeres during mitosis and meiosis. This association physically shields cohesin from phosphorylation by specific protein kinase(s). (ii) The presence of a specific protein phosphatase(s) at the centromeric region neutralizes the activity of the kinase(s). Recently, three independent studies published in *Nature *and *Developmental Cell *have convincingly demonstrated that enhanced dephosphorylation of centromeric proteins including cohesin may be a key mechanism responsible for centromeric cohesion of sister chromatids during meiosis and mitosis [15-17].

Using affinity protein purification followed by tandem mass spectrometry, the Nasmyth group found that protein phosphatase 2A (PP2A) subunits are the prevalent components that are co-purified in meiotic I cells of both fission and budding yeasts [16]. PP2A is known to exist primarily as a heterotrimeric complex which is composed of the scaffolding A subunit (PP2A-A), the variable regulatory subunit B (PP2A-B), and the catalytic subunit (PP2A-C). These investigators showed that despite the presence of subtypes for PP2A-B and PP2A-C subunits, only one subtype of PP2A-B (PP2A-B: Par1 in the fission yeast or Rts1 in the budding yeast) is found to be associated with Sgo1 [16,17]. Genetic experiments demonstrated that PP2A subunits (e.g., *Par1*^*B*'^, *Ppa2*^*C*^) are important for accurate chromosome segregation in fission yeast as their deletions results in moderate to severe missegregation of chromosomes; a detailed analysis revealed that chromosome missegregation occurs in meiosis II, but not in meiosis I, in fission yeast with either *Par1*^*B*' ^or *Ppa2*^*C *^deletion [16], a defect shared by the fission yeast with *Sgo1 *deletion. These observations thus suggest that PP2A, like Sgo1, may protect centromeric but not arm cohesion of sister chromatids. Consistent with this notion, fission yeast mutants with deletion of *Par1*^*B*' ^or *Sgo1 *are impaired in retaining centromeric Rec8 following meiosis I whereas deficiency in PP2A activity does not have an effect on expression as well as centromeric localization of Rec8 before the onset of anaphase I [16].

The mechanism by which PP2A protects centromeric cohesin is fully conserved in mammalian cells. Through a combination of co-immunoprecipitation and mass spectrometry, the Watanabe group identified PP2A as a major component in Sgo1, but not in control, immunoprecipitates [17], suggesting physical interaction between Sgo1 and PP2A. The direct interaction between Sgo1 and PP2A is subsequently confirmed by the yeast two-hybrid system. Although each PP2A subunit in mammalian cells has several subtypes/isoforms [18], only the PP2A-B subunit interacts with Sgo1 in human cells is PP2A-B'/B56 (including α, β, γ, δ, and ε isoforms) [17]. As the first step to study the functional significance of their interaction, these investigators examined the subcellular localization of PP2A. They observed that PP2A containing the B56α subunit localizes to the inner centromeres [17], thus consistent with the notion that PP2A may directly protect centromeric cohesion. Subsequent experiments demonstrated that depletion of PP2A through transfection with a specific small interfering RNA (siRNA) causes an enhanced rate of loss of centromeric cohesion coupled with mitotic arrest. However, the rate of loss of centromeric cohesion in PP2A-depleted cells is not high compared with that in Sgo1-depleted cells [17]. Although incomplete depletion of PP2A via RNA interference may partly be responsible for the poor penetrance of the phenotype, it is possible that other regulatory subunit may compensate for the absence of the PP2A-A subunit, which is directly targeted by a specific siRNA.

Similar results regarding PP2A's physical interaction with Sgo1 in human cells are also reported by the Yu group [15]. Co-immunoprecipitation analysis revealed that the interaction between PP2A and Sgo1 is enhanced during mitosis [15], which may be partly due to the fact that Sgo1 levels are higher in mitosis than during other phases of the cell cycle. Besides, these investigators showed that PP2A interacts efficiently with the N-terminus of Sgo1 that contains a coiled coil domain and that a single point mutation (Asn61Ile) abolishes their interaction. Again, siRNA experiments have been carried out to demonstrate PP2A's direct involvement in regulating centromeric cohesion. Depletion of PP2A results in chromosome missegregation that appears to be associated with loss of centromeric cohesion [15], a phenotype also observed in Sgo1- and Bub1-depleted cells [19].

## The kinase involvement

In eukaryotes, a hallmark for mitotic entry is the activation of mitotic kinases (e.g., Cdk1, Polo-like kinases, and Aurora kinases or their orthologues), resulting in phosphorylation of numerous mitotic targets essential for mediating mitotic progression. Early studies established that Bub1 is upstream of Sgo1 in the regulatory hierarchy [8,19]. Recent studies from both Nasmyth and Yu groups demonstrated that localization of PP2A to centromeres is dependent on Bub1 in yeast and human cells, respectively [15,16], indicating that PP2A lies downstream of Bub1. Consistently, in budding yeast, Bub1's localization at centromeres depends neither on Sgo1 nor PP2A [16]. However, the molecular mode of action is unclear regarding the role of Bub1 in regulation of PP2A and Sgo1. Given the fact that Bub1 is a protein kinase, it would be interesting to determine whether downstream components like PP2A and Sgo1 are direct substrates of Bub1. On the other hand, Plk1, a major mitotic kinase, is involved in phosphorylation of several downstream components of Bub1 during the metaphase-anaphase transition. (i) Plk1 directly phosphorylates SA2 in human cells, resulting in its dissociation from chromosome arms; (ii) Cdc5 (a Plk/Polo orthologue) and Plk1 phosphorylate Scc1 in budding yeast and human cells, respectively [20,21], facilitating its cleavage by separase; (iii) In *Drosophila*, Polo phosphorylates MEI-S332, which is essential for its removal from centromeres at anaphase [22]; (iv) Plk1 also phosphorylates several APC/C components [23] although its significance remains unknown.

Given the close involvement of Plk1/Polo in regulating anaphase entry and chromosome segregation, it is relevant to examine how Plk1 regulates PP2A activity, which in turn modulates centromeric cohesion. Interestingly, the Yu group showed that co-depletion of Plk1 and a key PP2A subunit through transfection of specific siRNAs causes retention of Sgo1 at centromeres in human cells, which is correlated with significant reduction of chromosomal missegregation; in contrast, co-depletion of Plk1 and Sgo1 fails to prevent missegregation of chromosomes [12,15]. Thus, these studies favor the possibility that the primary function of Plk1 may be to dislodge Sgo1 from centromeres when PP2A function is compromised. One caveat to this explanation is that Plk1 depletion alone does not enhance centromeric localization of Sgo1 [12,15,24] (Wang and Dai, unpublished data). Sgo1 is heavily phosphorylated during mitosis but not in interphase (Wang and Dai, unpublished data). Thus, it is of great interest to understand the molecular/biochemical basis underlying the close physical association between Sgo1 and PP2A, the latter being a protein phosphatase with a rather broad substrate spectrum. Apparently, PP2A does not cause dephosphorylation of Sgo1 during mitosis whereas other centromeric proteins such as SA2 and Scc1 are proposed to be dephosphorylated by the phosphatase.

## The regulatory hierarchy

The identification of PP2A as a new component in protecting centromeric cohesion of sister chromatids prompts additional studies determining its position in the regulatory hierarchy. As Sgo1 is primarily localized at centromeres during meiosis or early mitosis whereas PP2A signals are detected throughout the cell, it is possible that Sgo1 may help to recruit a specific form of PP2A to centromeres. This notion is consistent with the fact that most PP2A is not associated with Sgo1 [17]. Supporting this, the Nasmyth group showed that the amount of a centromere-bound PP2A subunit is greatly compromised in the fission yeast mutant with *Sgo1 *deletion during meiosis I, implying that the localization of PP2A to the centromeric region depends on Sgo1 [15]. On the other hand, studies from the Watanabe group demonstrated that the regulatory relationship between Sgo1 and PP2A is more complicated. (i) Localization of PP2A at centromeres is independent of Sgo1 whereas depletion of Sgo2 results in loss of centromeric PP2A during mitosis in human cells; (ii) once being centromere-localized, PP2A appears to be capable of conferring protection of centromeric cohesin in the absence of Sgo1 in both meiosis (yeast cells) and mitosis (human cells) [17]. Consistent with the latter observations, the Yu group showed that centromeric localization of PP2A is independent of Sgo1 in human cells [15].

**Figure 1:**
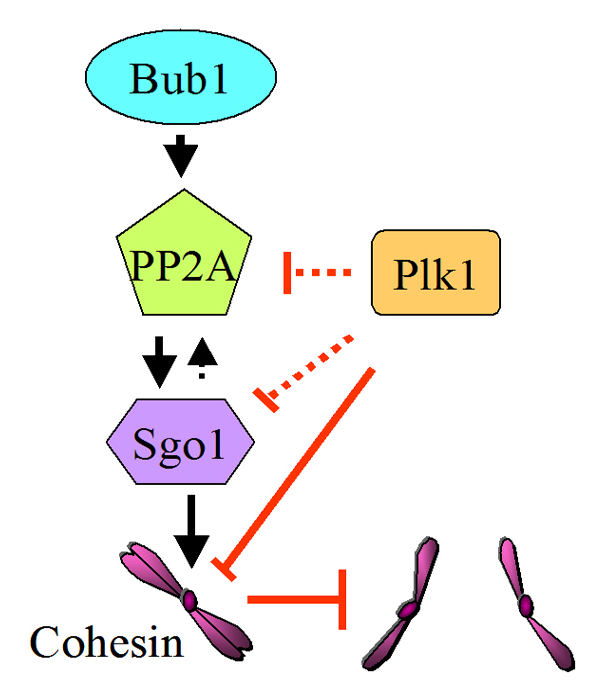
Key components in the regulation of centromeric cohesion of sister chromatids. Arrows (→) denote positive regulation and blocks (⊤) in red denote negative regulation. The dotted lines indicate that regulatory relationship has not been established experimentally.

Although it remains not entirely clear how Sgo1 regulates PP2A or vice versa with regard to protection of centrometic coehsin/cohesion during meiosis and mitosis, a simple model is proposed to illustrate the regulatory hierarchy in animal cells (Figure 1). Subcellular localization and/or activation of PP2A and Sgo1 depend on Bub1; PP2A is involved in recruiting to centromeres Sgo1, which may in turn have a positive feedback function through either stabilizing centromere-bound PP2A or facilitating its activation; centromeric PP2A and Sgo1 function to suppress phosphorylation of cohesin, thus positively regulating its function whereas Plk1 antagonizes the activities of PP2A and Sgo1, resulting in cohesin phosphorylation and its cleavage.
